# Development of cost-effective real-time PCR test: to detect a wide range of HBV DNA concentrations in the western amazon region of Brazil

**DOI:** 10.1186/1743-422X-11-16

**Published:** 2014-01-28

**Authors:** Alcione de Oliveira dos Santos, Luan Felipo Botelho Souza, Lourdes Maria Borzacov, Juan Miguel Villalobos-Salcedo, Deusilene Souza Vieira

**Affiliations:** 1 Fundação Oswaldo Cruz, Rondônia-FIOCRUZ, Porto Velho, Rondonia, Brazil; 2 Centro de Pesquisa em Medicina Tropical de Rondônia-CEPEM, Porto Velho, Rondonia, Brazil; 3 Universidade Federal de Rondonia-UNIR, Porto Velho, Rondonia, Brazil

## Abstract

**Background:**

Currently there is a significant risk of infection with hepatitis B virus (HBV) during blood transfusion in high epidemic area. This is due to the pre-seroconversion window period, immunovariant viral strains and the presence of occult HBV infection (OBI). The aim of this study was to develop an in-house real-time PCR-based method, which was both ultra-sensitive and efficient offering an alternative method for nucleic acid testing (NAT).

**Methods:**

A precore fragment with 109 bp was cloned and serial diluted to standard curve construction. The calibration of the HBV - DNA values was performed against OptiQuant® HBV-DNA Quantification Panel, Acrometrix Europe B.V.).

**Results:**

From our in-house plasmid we prepared serial dilutions ranging from 2 × 10^3^ – 2 × 10^9^ copies/ml. The threshold was adjusted automatically during analysis and the data collected were analyzed by linear regression (r^2^ = 0.99). The limit of detection for the assay with pHBVRO standards was 2000/ml in a total reaction volume of 30 μl. We found a strong correlation between the two methods (r^2^ = 0.9965 and p < 0.0001). The regression line give us the following equation: Log 10 (IU/mL) = 0.9038Log 10 (copies/mL) − 1.0643, suggesting that 1 IU/mL = 15 copies/mL.

**Conclusions:**

Therefore, we can affirm that the qHBVRO PCR can detect HBV DNA in individuals with hepatitis B at any stage of the disease showing high capacity for NAT screening in hepatitis b donors. This results of sensitivity could provide an advance for automation in blood banks and increasing safety of patients who receive blood transfusions.

## Introduction

The hepatitis B virus (HBV) is one of the most common human pathogens and can cause hepatitis and aggressive and advanced liver disease, including cirrhosis and hepatocellular carcinoma [1]. Despite the availability of a vaccine, the implementation of preventive measures and serological screening in blood banks remains a major public health problem worldwide [2]. HBV can be transmitted perinatally, percutaneously, sexually or by horizontal transmission, especially among children, presumably through open cuts or sores [3].

Early detection of HBV surface antigen (HBsAg) significantly reduces the risk of infection through blood transfusions [4]. However, there are two situations in the course of infection where this early detection is currently ineffective: First, during the acute phase of infection, there is a window period where HBsAg may be undetectable in serum [5]. In another situation, occult infection which is defined as the presence of HBV DNA in the liver (with or without detectable serum HBV DNA) may be present during the persistent of infection in subjects who test negative for hepatitis B surface antigen (HBsAg). These subjects often have very low viral load (< 200UI/ml) [6]. Reducing the risk of transmission in these situations will require increased sensitivity the detection of HBV surface antigen (HBsAg), screening for antibodies to HBV core antigen (anti-HBc), and continued testing and implementation of NATs [7,8].

Real-time polymerase chain reaction (qRT-PCR) has enabled the development of improved diagnostic tests offering greater speed while maintaining excellent levels of sensitivity and specificity [9-11]. qRT-PCR-based detection methods have been developed for the diagnosis of HBV and other pathologies in clinical laboratories [12-14].

To successfully monitor viral load, it is important to diagnose viral replication, establish the prognosis of liver disease, to assess the risk of disease progression, to identify patients who need antiviral therapy and to monitor the virologic response to treatment. Currently there are several types of detection and quantitation assays in use, with varying levels of success [15,16]. The aim of this study was to develop an in-house real-time PCR-based method, which was both ultra-sensitive and effective, offering a new NAT alternative.

## Materials and methods

### Clinical samples

This study included 134 patients with chronic HBV infection who were treated at the Viral Hepatitis Clinic Specialized Center of Research in Tropical Medicine in Rondonia (CEPEM). A control group of 30 donors, who all tested negative por ELISA for human immunodeficiency virus (HIV) 1 and 2, HBsAg, anti-HBc and anti-HCV, and who attended the blood bank of the State of Rondonia (FHEMERON) was included in the study. We also included 10 and 26 serum samples from individuals with chronic HCV and co-infection with HBV/HDV respectively.

### Ethical consent

This study was approved by the Brazilian Institutional Ethics Committee of the Centro de Pesquisa em Medicina Tropical (CEPEM), with process number 107/10. Written, informed consent was obtained from each patient for the publication of this manuscript and any accompanying images.

### DNA extraction

Viral DNA extraction was performed using the QIAamp DNA Mini Kit (Qiagen, Hilden, Germany) and 200 μl of serum according to the manufacturer’s instructions. Three samples with viral load known were tested: first with high viral load and the others medium and low viral load. After this were diluted with final volume 200 ul, 100 ul and 50 ul. Besides, HBV DNA was extracted from 22 samples of individuals with same profile serological: total isolated anti-HBc. These samples were diluted in 50 uL and 200 uL to optimize the final volume of extraction for samples with low viral load. Subsequently the samples were submitted to reaction for sensitivity analysis. Precipitated DNA was resuspended in elution buffer and stored at −20°C until further use. To avoid false-positive results, we followed strict procedures for nucleic acid amplification [17].

### In-house testing

Primer concentrations were optimized using a concentration gradient ranging from 100–900 nM and SYBR® Universal PCR Master Mix (Applied Biosystems, Foster City, CA, USA). TaqMan® probe concentrations were similarly optimized using a concentration gradient ranging from 50–300 nM.

### Ultra-sensitive real-time PCR

The assay was performed on an ABI 7500 platform (Applied Biosystems) with 30 μl reaction volumes containing 15 μl TaqMan® Universal Master Mix (Applied Biosystems), 3 μl HBVRO1 forward primer (5′-AGGAGGCTGTAGGCATAAATTGG 3′), 3 μl reverse primer (5′-GCACAGCTTGGAGGCTTG-3′), 0.6 μl probe (5′-FAM TCACCTCTGCCTAATC-3′-MGB, 6 μl extracted DNA and 2.4 μl of water.

### Construction of the standard curve

To construct the standard curve, we initially used conventional PCR with amplification of a 109 bp fragment in the pre-core region according Kavita 2006 adapted, selecting five samples with known viral load. Approximately 50 ng of DNA was used per reaction with a final volume of 50 μl. Amplification was performed on an ABI Prism 7500 Veriti (Applied Biosystems) with an initial denaturation temperature of 94°C for 5 min, followed by 40 cycles of 94°C for 1 min, 58°C for 45 sec, 72°C for 1 min and a final extension of 10 min at 72°C. The selected fragment was purified, ligated to the p-GEM-T Easy® vector (Promega, New York, USA), cloned into a prokaryotic system and subsequent linearization with *Pst*I (Invitrogen™ Life Technologies, Carlsbad, CA, USA). Absolute quantitation was used to determine the exact number of DNA molecules for estimating viral load.

### Inter- and intra-assay variation and reproducibility of real-time PCR

To determine intra-experimental variation, we tested the reproducibility of six HBV-positive sera with different viral loads, in duplicate, in the same reaction setup. The same set of samples was used in three experiments performed on different days, to estimate inter-experimental variation in the estimation of viral load. Reproducibility was estimated by calculating the coefficient of variation (CV), which is calculated as the ratio of the standard deviation and the mean of the replicates.

### HBV DNA quantification

Calibration curves for HBV DNA were constructed using the OptiQuant® HBV-DNA Quantification Panel, (AcroMetrix Europe BV). Specifically, a serial dilution was prepared from the standards included in the kit, ranging from 2 × 10^2^ – 2 × 10^6^ IU/ml. For our in-house plasmid, pHBVRO, a serial dilution was prepared with a range 2 × 10^3^ – 2 × 10^9^ copies/ml. The concentration was measured spectrophotometrically both by using a NanoDrop® ND-1000 (Thermo Scientific NanoDrop Products, Wilmington, Delaware), and the measurements were recorded in units of nanograms per microliter, which was converted into copies per microliter by using the following equation: ([x ng/μL × 10^-9^] / [p-GEM-T Easy® vector and 109 HBV DNAbps × 660]) × 6.022e^23^ = y copies/μL. Using linear regression a standard curve was constructed, which was used to convert copies/ml to standard international units (IU/ml).

### Quantification panel

To compare the performance of our in-house method (qHBVRO) with that of the commercial kit, OptiQuant® HBV-DNA Quantification Panel (AcroMetrix® Europe BV), we tested 100 serum samples collected from patients chronically infected with HBV.

### Statistical analysis

The correlation between the AcroMetrix® test kit and the qHBVRO assay was calculated using GraphPad 5.0 (GraphPad software) and a two-tailed Pearson′s correlation test with a confidence interval of 95%. The units for measuring viral load (copies/ml and IU/ml) were transformed to log base 10.

### Analytical specificity

We tested 30 samples from blood donors, 10 serum samples from mono-infected HCV patients, 28 samples from patients co-infected with HBV/HDV and 15 samples that were judged indeterminate for HBV surface antigen by the Serology Laboratory of Viral Hepatitis Clinic - IPEPATRO. All samples were submitted to qHBV PCR to determine viral load.

### Analytical sensitivity

We selected 15 sera, which were considered indeterminate for HBsAg by ELISA when tested in duplicate and which had absorbance values within the gray zone, or ± 10% cut-off confidence interval. These samples were subjected to three separate assays, with each sample performed in duplicate to evaluate the performance of our assay in detecting uncertain samples.

## Results

### Analytical sensitivity and efficiency

**Figure 1:**
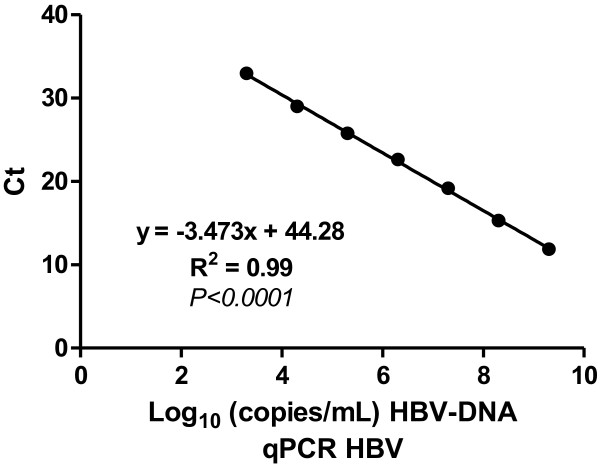
qHBVRO standard curve as determined by linear regression.

From our in-house plasmid we prepared serial dilutions ranging from 2 × 10^3^ – 2 × 10^9^ copies/ml. The concentration of primers used was 300 nM, for both primers, and 100 nM for the probe. The threshold was adjusted automatically during analysis and the data collected were analyzed by linear regression (r^2^ = 0.99), Figure 1. The limit of detection for the assay with pHBVRO standards was 2000/ml in a total reaction volume of 30 μl. Two positive samples with known viral load were used as internal controls. HBV DNA extraction was patterned on elution of 200 μl because there was no significant variation in sensitivity of method in different volumes of patients with intermediate and high viral load. But the 22 samples of patients with serological profile “anti-HBc total isolated” eluted in 50 and 200 uL, 15 samples were negative on both elution, 5 samples were positive only in elution of 50 uL within which 2 samples were positive in both elution.

### Inter- and intra-assay variation and reproducibility of real-time PCR

The six HVB-DNA positive sera tested showed no statistical differences between repeats. The coefficient of variation was similar in both high and low viral load samples (0.01-0.16%), indicating the same efficiency of amplification for varying viral loads. There was no statistical difference in intra-and inter-assay variation (CV), which confirmed the reproducibility of the assay (Tables 1 and 2).

**Table 1:** Intra-experimental variation in the qHBVRO assay for serum samples

**qHBV RO - intra assay**						
**Sample**	**1st run (IU/mL)**	**2nd run (IU/mL)**	**3th run (IU/mL)**	**Avarage inter assay**	**SD**	**CV**
1	4.6 × 10^6^	4.6 × 10^6^	4.6 × 10^6^	4.6 × 10^6^	0.027 × 10^6^	0,01
2	2.8 × 10^4^	2,7 × 10^4^	2.7 × 10^4^	2.7 × 10^4^	0.025 × 10^4^	0,01
3	5.0 × 10^2^	4.3 × 10^2^	4.6 × 10^2^	4.6 × 10^2^	0.36 × 10^2^	0,08
4	3.2 × 10^2^	2.9 × 10^2^	2.7 × 10^2^	3.0 × 10^2^	0.26 × 10^2^	0,09
5	3.3 × 10^5^	3.1 × 10^5^	3.0 × 10^5^	3.2 × 10^5^	0.16 × 10^5^	0,05
6	4.8 × 10^4^	4.0 × 10^4^	4.3 × 10^4^	4.4 × 10^4^	0.38 × 10^4^	0,09

**Table 2:** Inter-experimental variation in the qHBVRO assay for serum samples

**qHBV RO - inter assay**						
**Sample**	**1st run (IU/mL)**	**2nd run (IU/mL)**	**3th run (IU/mL)**	**Avarage inter assay**	**SD**	**CV**
1	4.6 × 10^6^	5.0 × 10^6^	4.6 × 10^6^	4.7 × 10^6^	0.02 × 10^6^	0,05
2	2.7 × 10^4^	3.4 × 10^4^	27 × 10^4^	2.9 × 10^4^	0.004 × 10^4^	0,14
3	4.3 × 10^2^	4.6 × 10^2^	4.0 × 10^2^	4.3 × 10^2^	0.3 × 10^2^	0,07
4	3.2 × 10^2^	2.7 × 10^2^	3.0 × 10^2^	3.0 × 10^2^	0.2 × 10^2^	0,08
5	3.3 × 10^5^	3.0 × 10^5^	2.8 × 10^5^	3.0 × 10^5^	0.2 × 10^5^	0,08
6	5.0 × 10^4^	4.9 × 10^4^	4.0 × 10^4^	4.6 × 10^4^	0.5 × 10^4^	0,11

### Validation of the qHBVRO method

**Figure 2:**
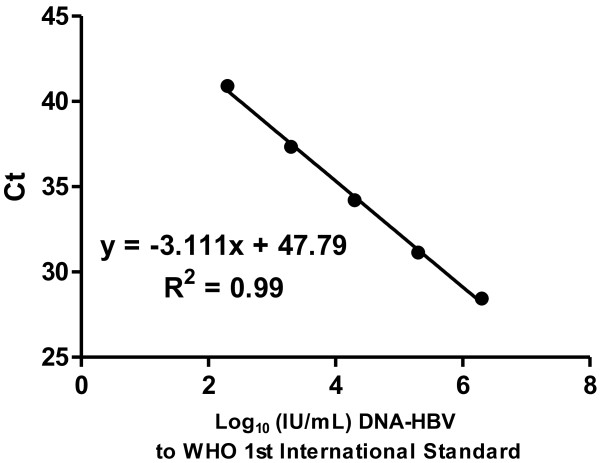
WHO 1st International Standard (HBV ® kit Acrometrix DNA) as determined by linear regression.

**Figure 3:**
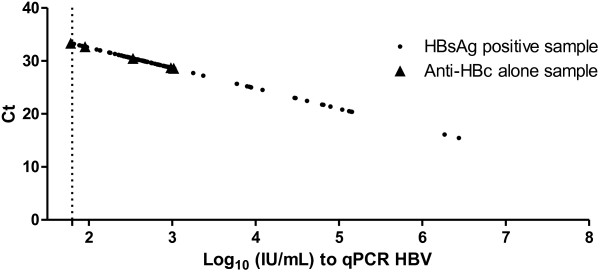
**Analytical sensitivity of qPCR HBV demonstrated with 97 samples: 91 samples positive for HBsAg and 6 samples total anti-HBc isolated.** These 6 samples were DNA-HBV positive detected within 50 clinical samples tested in patients with profile total anti-HBc isolated. The dashed line is the limit of analytical detection of qPCR HBV.

Using linear regression, we found a strong correlation between the qHBVRO assay and the AcroMetrix® HBV-DNA kit (r^2^ = 0.998 and p <0.0001) as shown in Figure 2. Viral load values between the AcroMetrix® HBV-DNA and the qHBVRO assay were compared for 134 patients by Pearson’s correlation. We found a strong correlation between the two methods (r^2^ = 0.9965 and p < 0.0001). The regression line give us the following equation: Log 10 (IU/mL) = 0.9038Log 10 (copies/mL) − 1.0643, suggesting that 1 IU/mL = 15 copies/mL (Figure 3).

### Analytical specificity and detection performance of indeterminate for ELISA samples and anti-HBc isolated samples

**Figure 4:**
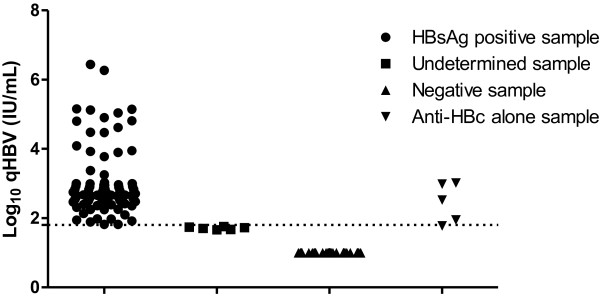
**Analytical specificity of samples that tested positive, undetermined, negative and anti-HBc alone by ELISA using the qPCR HBV *in house*.**

Of the 15 indeterminate for ELISA samples tested, six were positive for HBV DNA by both methods (Table 3). All samples that were classified as negative by ELISA were confirmed as such as shown. Of the 22 samples isolated anti-HBc eluted in 50 uL, 5 were positive (Figure 4).

**Table 3:** Data summary for indeterminate samples

	1st run (Ct*)	2nd run (Ct*)	3th run (Ct*)	**Mean**	**SD**	**CV**	**Log**_**10 **_**IU/ml**	DO*	**Cut-off**
1	37.31	37.56	37.74	37.54	0.21595	1%	0.69	0.086	0.062
2	38.90	38.98	39.1	38.99	0.10066	0%	0.31	0.075	0.079
3	38.69	38.83	38.95	38.82	0.13013	0%	0.35	0.070	0.063
4	37.47	37.54	37.87	37.63	0.21362	1%	0.66	0.062	0.065
5	39.05	39.1	39.45	39.20	0.21794	1%	0.25	0.060	0.068
6	38.94	39.03	39.26	39.08	0.16503	0%	0.28	0.069	0.068

DO*: Optical Density by spectrophotometry (ELISA).

Ct*: Amplification cycle in the assay qHBVRO.

## Discussion

Currently, there is a significant risk of infection with hepatitis B virus (HBV) during blood transfusions. This is due to the pre-seroconversion window period, immunovariant viral strains and the presence of occult HBV infection (OBI) [8,18,19]. Combined detection of HBsAg and anti-HBc constitutes an important strategy in donor screening which excludes the vast majority of OBIs [20-22]. However, in many countries, especially in areas of low HBV prevalence (< 3%) these strategies are inefficient especially during the pre-seroconversion window period. However, in countries with high prevalence of positivity for anti-HBc, using this marker could involve both the exclusion of several potential donors leading to a decrease of blood products in various regions - such as the failure to block potential donors with OBI - keeping present the risk of post-transfusion HBV [18,23]. The qHBVRO test presents an excellent alternative to HBV detection and quantification because it allows significant reduction in the risk of transmission during the window period as well as improved detection of occult HBV.

In this study we developed a qRT-PCR assay for the identification and quantification of HBV DNA with an efficiency of 94.06% and good correlation with the currently used commercial test: WHO 1st International Standard (HBV ® kit Acrometrix DNA), r = 0.998, p < 0.0001. The qHBVRO PCR test was 100 fold more sensitive, allowing detection of up to two thousand copies per ml of serum in HBV-infected individuals. Our method also proved to be more sensitive than other in-house qRT-PCRs [24-26], and could detect occult hepatitis B infections as well as cases which were inconclusive by ELISA, using only 6 μl of DNA extracted from 200 μl of serum in final reaction volume of 30 μl for qHBVRO. However, was observed that in cases of samples with low viral load is important to consider a smaller volume in the elution of DNA. It is the case of 22 samples tested from individuals with isolated anti-HBc where 5 were positive in 50 uL elution and only 2 were positive on PCR qHBV. These results reinforce the importance that in case of occult infection or immunological window period, the concentration of DNA is an important factor to consider.

The high analytical specificity of the test, using samples from individuals that tested positive, negative and indeterminate for HBsAg by ELISA demonstrates that qHBVRO PCR can detect HBV DNA in individuals with hepatitis B at any stage of the disease, qualifying it as an important alternative NAT. The qHBVRO assay is highly reproducible, with low intra- and inter-experimental variation of between 0-1% (CV), whereas other in-house tests, which are considered to have good reproducibility, show experimental variation of 4.94–10.59% [27-31].

Our method proved to be efficient, sensitive, specific and reproducible in the detection of occult HBV, and could therefore be used for nucleic acid testing (NAT) in blood banks to prevent HBV transmission by blood transfusion. The advantages of NAT relating to cost and effectiveness compared with serological diagnostics have been widely debated [28-30]. It has been suggested that NAT offers advantages in many instances including occult infections, in the confirmation of viremia, for screening blood- and organ-donors, discriminating between patients with chronic or acute infection which has been resolved, diagnosis of perinatal infection, solving indeterminate serological results, monitoring patients on antiviral therapy and to identify the virus in immunocompromised individuals [31-33].

## Conclusion

In conclusion, the real-time PCR assay qHBVRO is appropriate for the quantification of HBV DNA in serum samples. This test is reproducible and proved be sensitive detecting samples with low viral load. Therefore, we can affirm that the qHBVRO PCR can detect HBV DNA in individuals with hepatitis B at any phase of disease showing good NAT screening for hepatitis B. Samples of patients anti-HBc positive isolated were selected and submitted to qHBVRO test to enhancing sensitivity the this results. This developed test may be automated and used in blood banks, increasing safety of patients who receive blood transfusions.

## Competing interests

The authors declare that they have no financial or competing interest with this article.

## Authors’ contributions

AOS participated in the design of the study, drafted the manuscript and in its design and coordination. LFBS participated in the PCR amplification and sequencing process. DSV participated in the design of the study. LMB participated in the elaboration of the manuscript. JMS participated in the design of the study. All authors read and approved the final manuscript.
